# In Vitro Evaluation of the Permeability of Different Resorbable Xenogeneic Membranes after Collagenolytic Degradation

**DOI:** 10.3390/membranes12080787

**Published:** 2022-08-17

**Authors:** Ramona Kölliker, Stefan P. Hicklin, Constanze Hirsiger, Chun Ching Liu, Fredi Janett, Patrick R. Schmidlin

**Affiliations:** 1 Clinic of Conservative and Preventive Dentistry, Center of Dental Medicine, University of Zurich, Plattenstrasse 11, 8032 Zurich, Switzerland; 2 Clinic of Reproductive Medicine, Vetsuisse-Faculty, University of Zurich, Winterthurerstrasse 204, 8057 Zurich, Switzerland

**Keywords:** membrane, guided tissue regeneration, collagen, collagenolytic activity, bacteria

## Abstract

In this in vitro study, we compare the penetration of cells through different resorbable collagen membranes, which were collagenolytically degraded over different time periods. Three different resorbable collagen membranes were evaluated, including two non-cross-linked (NCL) membranes—namely, a porcine (NCL-P) and an equine (NCL-E) membrane—and an enzymatically cross-linked porcine (ECL-B) membrane. A special two-chamber model was fabricated, allowing for the placement of separating membranes, and a non-porous polyester membrane was used as a negative control (C), in order to verify the impermeability of the experimental chamber device. Round membrane samples with a diameter of 16 mm were fabricated. Eighteen membranes of each type were punched and placed on polyethylene nets as carriers. The membranes were then biodegraded—each on its carrier—in 12-well polystyrene plates: three samples of each membrane type were degraded for 1.5, 3, 6, or 12 h in 2 mL of a buffered collagenase solution, at 37 °C. For control purposes, three samples of each membrane type were not degraded, but only immersed in buffer solution for 1.5, 3, 6, or 12 h, at 37 °C. Another three samples of each type of membrane were degraded until complete dissolution, in order to determine the full hydroxyproline content for comparison. Liquid-preserved boar semen (containing at least 120 million sperm cells per milliliter) was used to test the cell occlusivity of the degraded membranes. At baseline and initial degradation, all tested membranes were tight, and no penetration was observed with up to 30 min of incubation time (results not shown). After 1.5 h, cells were partially capable of penetrating the NCL-E membrane only. One sample showed leakage, with a sperm volume of 1.7 million cells/mL over all samples. No penetration occurred in the test, NCL-P, and ECL-B groups. After a degradation time of 3 h, the NCL-P and ECL-B membranes remained occlusive to cells. All the membranes and measurements indicated leakage in the NCL-E group. After 6 h, four NCL-P measurements showed the first signs of cell penetration, as boar spermatozoa were detectable in the lower chamber (64 million cells/mL). The ECL-B membranes remained completely cell occlusive. After 12 h, four NCL-P measurements were cell penetration positive (14.6 million cells/mL), while the ECL-B group remained tight and showed no cell penetration. As the findings of our study are well in accordance with the results of several previous animal studies, it can be concluded that the surrogate model is capable of performing rapid and cheap screening of cell occlusivity for different collagen membranes in a very standardized manner. In particular, claims of long degradation resistance can be easily proven and compared. As the boar spermatozoa used in the present report had a size of 9 × 5 μm, smaller bacteria are probably also able to penetrate the leaking membranes; in this regard, our proposed study set-up may provide valuable information, although it must be acknowledged that sperm cells show active mobility and do not only translocate by growth.

## 1. Introduction

The principles of guided tissue regeneration (GTR) and bone regeneration (GBR) have been well investigated and established in the field of dentistry [1,2]. The original principle relies on the use of occlusive membranes to establish covered and protected defect areas, separating them from fast-growing unspecific cell populations of the adjacent soft tissues (e.g., fibroblasts or epithelial cells).

An ideal barrier membrane should possess biocompatibility and provide cell occlusivity, combined with appropriate clinical manageability, space maintenance, and the possibility to resorb gradually over time, in order to avoid a follow-up surgery for membrane removal [3]. At present, a variety of resorbable and non-resorbable membranes are available on the market. Collagen-based membranes have several advantages over non-resorbable membranes, such as easier clinical handling, no need for membrane removal, lower patient morbidity, and fast resorption in the case of wound dehiscence and membrane exposure, therefore improving soft tissue healing [4]. Fast resorption and degradation of membranes has been shown to lead to rather unfavorable healing [5,6]. In order to obtain adequate regeneration, membranes should maintain their cell-occlusive properties for at least four weeks [7,8,9]. This feature is strongly linked to the porosity of the membrane, which reflects a double-edged sword in its practical sense: on one hand, a good porosity allows for the diffusion of nutrients, oxygen, and bioactive substances, which are important for the healing process and vascularization; on the other hand, if the pores exceed a certain critical size and number, an early ingrowth of undesired cells or even bacteria in the area to be regenerated may occur, which may have a significant negative impact on the early healing and differentiation processes, especially in the first four weeks [10,11]. In particular, in cases with wound dehiscence and exposure of the collagen membrane, the degradation of the membrane may be accelerated, as caused by the activity of macrophages and neutrophils, which has negative impacts on membrane stability and cell occlusiveness [11,12]. Even without membrane exposition and a more or less uncontrollable degradation and inflammation processes, the degradation data for collagen membranes are quite heterogenous. Studies have shown the first signs of degradation starting from day four up to six weeks after incorporation of the membrane, depending on the type of membrane, the origin and type of the collagen, as well as the manufacturing process (e.g., cross-linking or physical treatment with pressure or temperature) [13,14,15,16]. The durability and quality of the barrier function remain difficult to determine and/or predict, both in the lab and clinically.

As the main key factor for successful tissue regeneration remains the predictable establishment of stable, protected, and separated surgical compartments for at least four weeks, it therefore remains essential to focus on the evaluation of membrane degradation patterns and the duration of the desired barrier function in vitro and in vivo using reliable and easy-to-apply standardized and comparative methods. The assessment of cell proliferation on and through membranes in this context remains a challenge. While the proliferation and differentiation of various cell types on membranes surface has previously been well-studied, in terms of simulated biocompatibility evaluations, methods for the evaluation of the penetration capability through membranes are still scarce [17,18,19]. As cell growth under laboratory conditions is complex and time-consuming, while animal studies are expensive and raise additional ethical concerns, the use of simple surrogate cell assays to evaluate membrane permeability and occlusive capacity is still of great interest. Under this background, a new model has previously been introduced, in which bull and boar spermatozoa and fluorescent beads were used to assess and compare the cell occlusion potential of membranes [20]. This method allowed for convenient and rapid testing of cell and particle penetration, providing a solid basis for the screening of dental membranes after being exposed to different degradation durations and methods; however, the latter aspect was not considered in the previous study.

Therefore, the goal of this follow-up study is to assess and compare the cell permeability using xenogeneic sperm cells and three different collagen membranes after standardized in vitro degradation at different time points. We hypothesized that the impermeability of a collagen membrane depends on its individual degradation behavior, which results in respective collagen structure changes during the collagenolytic challenge. As a primary outcome parameter, the penetration of boar cells from one (cell-containing) compartment to another (cell-free) one through the tested membranes was chosen. As a secondary outcome parameter, hydroxyproline dissolution in the collagenolytic challenge was determined. In addition, SEM images were captured to depict the membrane morphology in this context.

## 2. Materials and Methods

### 2.1. Membranes and Degradation

Three different resorbable collagen membranes were evaluated in this in vitro trial. We used two non-cross-linked (NCL) membranes—a porcine (NCL-P) (Geistlich Bio-Gide^®^, Geistlich Pharma AG, Wolhusen, Switzerland) and an equine (NCL-E) membrane (Parasorb Resodont^®^ Forte, Resorba Medical GmbH, Nürnberg, Germany)—as well as a cross-linked porcine (ECL-B) membrane (Ossix^®^ Plus, Datum Dental Ltd., Lod, Israel). In order to cross-link the collagen molecules, naturally occurring non-toxic sugar was used.

**Figure 1:**
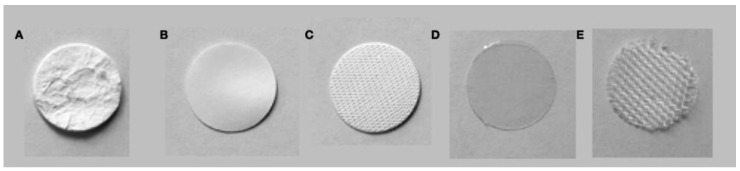
Samples of the test membranes ((**A**) NCL-P; (**B**) NCL-E; (**C**) ECL-B), the non-porous polyester membrane (**D**), and the carrier mesh (**E**).

A non-porous polyester membrane (PR–P 100 MY, Folex AG^®^, Seewen, Switzerland) served as a negative control (C), in order to verify the impermeability of the experimental apparatus (Figure 1).

Round membrane samples with a diameter of 16 mm were fabricated. Eighteen membranes of each type were punched and placed on polyethylene nets (screen insert Ø 45 mm PE-HD; Faust Laborbedarf AG, Schaffhausen, Switzerland) serving as carriers. 

The membranes were then biodegraded (each on its carrier) in 12-well polystyrene plates (Thermo Scientific™ Nunc™ Cell-Culture Treated Multidishes, Thermo Fisher Scientific, Rochester, NY, USA). Three samples of each membrane type were degraded for 1.5, 3, 6, or 12 h in 2 mL of a buffered collagenase solution, at 37 °C. The bacterial collagenase solution was derived from *Clostridium histolyticum* Type IA C9891 (Sigma-Aldrich Co. LLC, St. Louis, MO, USA), which was added (2 mL) to the Tris-buffer of 0.1 M TRIS-HCl with CaCl_2_ to reach a collagenase concentration of 8 U/mL. In order to halt biodegradation, the collagenase was inactivated through the addition of 2 mL of 17% EDTA (Ethylenediaminetetraacetic acid; Kantonsapotheke Zürich, Schlieren, Switzerland). Finally, all samples were washed with phosphate-buffered saline solution (PBS) (Oxoid™, Thermo Fisher Scientific, Rochester, NY, USA). 

For control purposes, three samples of each membrane type were not degraded, but only immersed in buffer solution for 1.5, 3, 6, or 12 h, at 37 °C. Another three samples of each type of membrane were degraded until complete dissolution, in order to determine the full hydroxyproline content for comparison. 

### 2.2. Cell Permeability Testing 

Liquid-preserved boar semen was used for this experiment (SUISAG, Sempach, Switzerland), containing at least 120 million sperm cells per milliliter. The cells were diluted and preserved in XCell media (IMB Technologies, L’Aigle, Switzerland), at 18 °C, until used. Sperm concentration and motility were determined using a computer-assisted sperm analysis (IVOS II, Version 1.5, Hamilton Thorne Biosciences, Beverly, MA, USA), which also allowed for the tracking of spermatozoa trajectories. 

**Figure 2:**
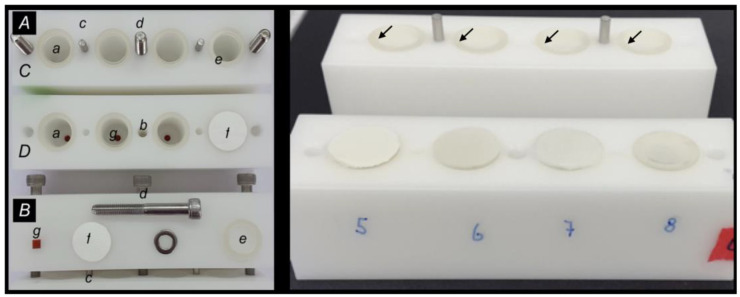
**Left**: Disconnected two-chamber system model: View from above ((**A**) with the upper (**C**) and lower component (**D**)) and the side ((**B**) upper part). The models had four chambers each (a). Holes (b) were drilled in the blocks, which allowed for firm connection and anchorage using metal guidance pins (c) and fixation screws (d). Rubber rings (e) were used to seal the individual chambers between the upper and lower blocks. The membranes on carriers (f) were placed between the blocks and rubber rings, respectively. Rubber plugs (g) at the top and bottom closed the chambers. **Right**: Test membranes and foil in situ. Rubber rings placed on the upper block (arrows) to ensure a tight seal and fixation of the membranes.

A custom-made two-chamber system, which has been validated in an earlier study [20], was used to monitor the tightness or penetration of the cells. It consists of an upper and a lower chamber, which could be separated by the test membranes. The chamber floors had additional access holes, allowing for liquid application and sampling at any desired time (Figure 2).

For the experiment, the lower chamber was loaded with 2 mL of the cell media, and the membrane samples were carefully placed on elastic O-rings on the lower chamber; both chambers were then tightly fixed together using screws. Afterwards, the upper chamber was filled, through the access hole, with sperm-containing XCell media and tightly closed with custom-made rubber plugs. The two-chamber system was then incubated, at 37 °C, for 10 min or 30 min, until cell measurements took place. For this purpose, a sperm cell solution sample was retrieved from both chambers and evaluated by phase-contrast microscopy using a computer-assisted sperm analysis software (IVOS II, Hamilton Thorne Biosciences, Beverly, MA, USA). The samples from the lower chambers were evaluated for sperm cell concentration, in order to evaluate the membrane permeability for boar sperm cells, and the pore size. Samples from the upper chambers were checked to monitor the continuous motility for uninhibited sperm cell vitality. 

### 2.3. Evaluation of the Collagen Content

The collagen content of the degraded membranes was determined in 100 µL of the original degradation solution of each sample in duplicate. For this purpose, the hydroxyproline dissolved from the membranes was stained red using a hydroxyproline assay kit MAK008 (Sigma-Aldrich Co. LLC, St. Louis, MO, USA), then measured quantitatively at 560 nm using a SpectraMax^®^ M2e (Molecular Devices, LLC, San Jose, CA, USA). The amount of hydroxyproline released from each sample was related to the total hydroxyproline content of the respective type of membrane, and the calculated value given as a percentage.

### 2.4. Scanning Electron Microscopy

Finally, the degraded and perfused membranes were visually assessed using scanning electron microscopy (SEM), in order to evaluate the surface structure alterations due to collagen degradation and sperm cell penetration at the different time periods. 

Samples were fixed for 24 h in 2.5% glutaraldehyde solution, then rinsed with PBS and dehydrated twice for 15 min in ascending concentrations of ethanol (50%, 70%, 80%, and 90%, respectively). The membranes were then immersed three times for 15 min in 94% and 60 min in 100% ethanol, respectively. Finally, samples were subjected to critical point drying (Bal-Tec CPD030, Balzers, Liechtenstein). All the samples were then cut in the middle and mounted on SEM mounts (Bal-Tec AG, Blazers, Liechtenstein), with each cut shown on the side of the membrane (i.e., upside-down and vice versa). Samples were gold sputtered (Balzers SCD 030, Balzers Union, Balzers, Liechtenstein) for 60 s in an argon gas atmosphere at a target distance of 50 mm and pressure of 5 Pascal (Pa) at 45 mA. SEM images (Supra 50 VP FESEM, Carl Zeiss, Oberkochen, Germany) were taken at a working distance of 9.2 mm, an acceleration voltage of 10 kV, and a magnification of 5000×. 

### 2.5. Analysis

Data are merely descriptively presented; that is, mean values and standard deviations from the triplicate measurements are provided. The results of the SEM evaluation are also qualitatively described.

## 3. Results

### 3.1. Membrane Degradation 

**Figure 3:**
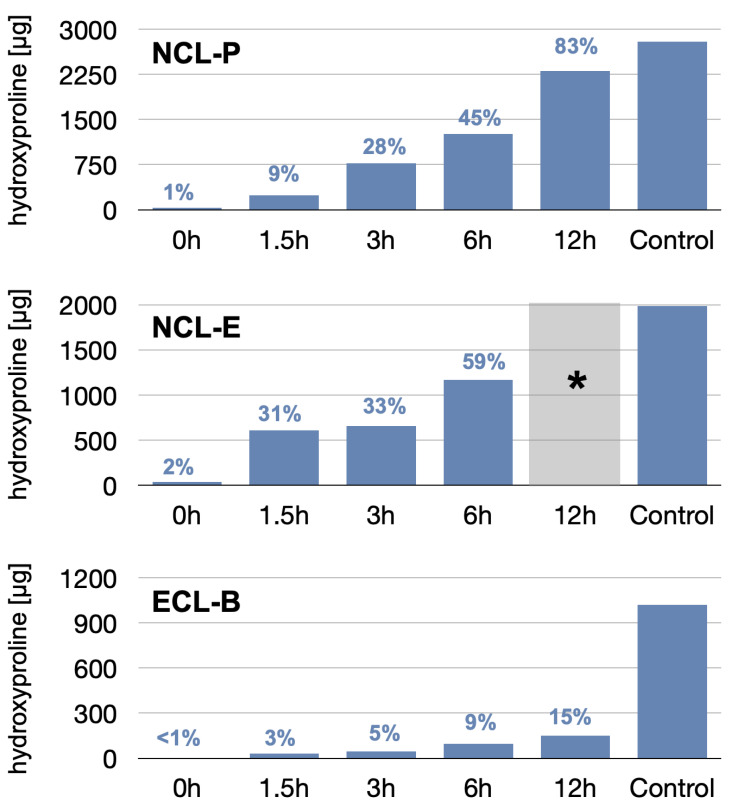
Results of the hydroxyproline (HP) concentration measured in the immersion/degradation solutions. Control values represent the potentially available HP concentrations determined in three completely dissolved control specimens of each material. The percentages represent the relative mean HP dissolution, with reference to the control, after different dissolution times. (* Note: No measurements could be performed for NCL-E after 6 h, as the membrane had completely disintegrated and was not usable).

The results of the membrane dissolution test are depicted in Figure 3. As a first result, we observed that the hydroxyproline content of the three different membranes (controls; i.e., completely degraded membranes) already varied significantly, with values of 1021 µg, 1984 µg, and 2792 µg, for the NCL-P, NCL-E, and ECL-B membranes, respectively, due to difference in material density, thickness, and architecture. 

The relative dissolution also varied between the membranes. The least dissolution was observed for the ECL-B membrane, reaching a maximal dissolution after 12 h accounting for 150 µg (i.e., 15% of the total hydroxyproline amount), showing a slow, almost linear increase over time. The NCL membranes showed both faster and higher degradation values, where the fastest dissolution was found for the NCL-E group. At 3 h, both NCL membranes showed comparable relative degradation, around 30%.

While the NCL-E membrane was already degraded to such an extent after 6 h that handling with regard to cell penetration was no longer possible, the NCL-P membrane still showed sufficient mechanical manipulability after 12 h, despite a dissolution of more than 83%.

### 3.2. Sperm Cell Penetration

**Figure 4:**
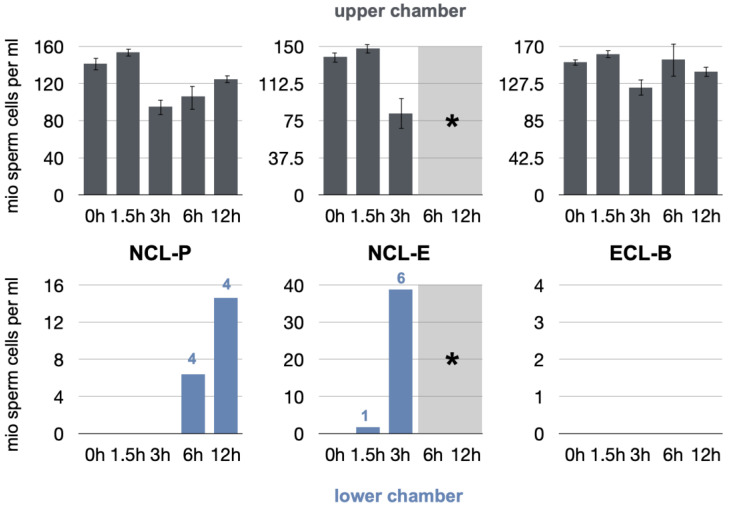
Results of the sperm cell counting (millions per milliliter) after different degradation times (bar charts with S.E. in the upper chamber). Results of the triplicate measurements after 10 and 30 min are given for the upper and lower chambers separately. The numbers given in the lower chamber indicate the actual number of leaking specimens (* Note: No measurements could be performed for NCL-E after 6 h, as the membrane had completely disintegrated and was not usable).

The results of the sperm cell penetration experiments are depicted in Figure 4. 

Viable boar spermatozoa were measurable in the upper compartments of the test apparatus throughout the experiments (>80 million cells/mL). The cells and their mobility and vitality were, therefore, not affected by temperature changes, chemical residues on the membranes, or the handling required to perform the tests, among other factors. The negative control group proved the chamber to be completely sealed, as no spermatozoa were able to penetrate into the lower chamber of the test apparatus throughout the experiment (results not shown).

At baseline and initial degradation, all the tested membranes were tight, and no penetration was observed, up to 30 min of incubation time (results not shown). 

After 1.5 h, cells were partially capable of penetrating through the NCL-E membrane only. One sample showed leakage, with a sperm concentration of 1.7 million cells/mL. No penetration occurred in the test NCL-P and ECL-B groups. 

After a degradation time of 3 h, the NCL-P and ECL-B membranes remained occlusive to cells, while all membranes and measurements indicated leakage in the NCL-E group. The mean sperm concentration increased, accounting for 38.8 million cells/mL. With regard to the following observation periods, the latter membrane could not be tested any more, as the membranes had structurally disintegrated and more or less completely dissolved, and could not be manipulated despite the mesh. 

After 6 h, four NCL-P measurements showed first signs of cell penetration, as boar spermatozoa were detectable in the lower chamber (64 million cells/mL). The ECL-B membranes remained completely cell occlusive.

After 12 h, again, four NCL-P measurements were cell penetration-positive (14.6 million cells/mL). The ECL-B group remained tight and showed no cell penetration.

### 3.3. SEM Evaluation

**Figure 5:**
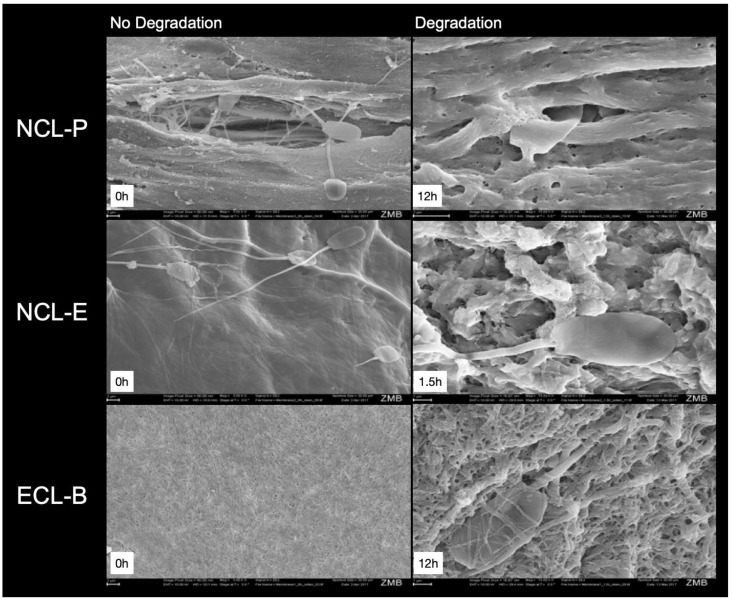
Representative SEM images of the test membranes without degradation (**left**; magnification, 5000×) and after degradation (**right**, 15,000×; 1.5 h for NCL-E and 12 h for NCL-P and ECL-E). Please note the sperm cells, which are visible and trying to pass through the collagen fibers and different pores.

Representative images of the membranes, illustrating the different degradation degree of the membranes, are shown in Figure 5. 

All the membranes showed an even, tight surface at baseline, where the NCL-E membrane showed an especially smooth and tight surface. However, after 1.5 h, this membrane already showed an uneven appearance and no distinct but, instead, a rather loose and porous fiber pattern. 

At baseline, the NCL-P membrane showed a smooth but, at the same time, uneven (and, in some parts, porous) structure. After 12 h, the collagen fibers are clearly visible at higher magnification, including some pores. 

The ECL-B membranes showed a fibrous surface and interlinking of the fibers, which still seemed to be more or less unchanged, even at higher magnification, after 12 h. The fibers and pores looked smaller, when compared to the other membranes.

## 4. Discussion

Native collagen usually undergoes rapid degradation by tissue-specific proteases, collagenases, and macrophages [21]. To slow down the degradation process, collagen can be stabilized by cross-linking. Several animal and human studies have shown that cross-linked collagen membranes are more resistant against degradation than non-cross-linked membranes [15,22,23,24,25]. The present laboratory study corroborated these findings, as the cross-linked membrane displayed a delayed degradation behavior, compared to native collagen membranes, and remained stable and occlusive over the whole observation period. For comparison, after 12 h of degradation, the cross-linked membranes (ECL-B) showed a collagen reduction of roughly only 15%, while that in the native collagen membranes was 59% and 83% for NCL-E and NCL-P, respectively. Under pre-clinical conditions, a histological study in rats has also shown almost complete resorption of a native collagen membrane (NCL-P) after four weeks and complete vascularization of the membrane after only two weeks of healing, whereas the cross-linked membrane (NCL-B) showed first signs of resorption and vascularization only after 24 weeks of healing [13].

The actual degradation speed and modality also depend on the defect and healing conditions, as well as, probably, on the actual animal model and individual healing characteristics. However, distinct dissolution patterns persist. Another animal study in dogs considering critical size alveolar ridge defects has also investigated the degradation pattern of cross-linked membranes (ECL-B) and native collagen membranes (NCL-P) [22]: eight weeks after extraction of the pre-molars in the mandible, the membranes were inserted with and without bone augmentation. After 8 weeks of submerged healing, the native collagen membrane showed little signs of degradation and solely isolated tears. After 16 weeks of healing, progression of the degradation was visible. After 24 weeks of healing, the findings were still heterogenous, as the degradation was moderate in some animals while, in others, the membranes had been completely degraded. In contrast, the applied cross-linked membranes showed only moderate degradation after 24 weeks. 

Interestingly, in the present study, the two tested native collagen membranes also showed obvious differences in their biodegradation pattern. These differences, including different hydroxyproline dissolution kinetics, may also be present in membranes of the same origin (e.g., porcine-derived membranes). In this context, Bozkurt et al. [26] have shown, in a combined in vitro and in vivo study, that the amount of hydroxyproline was significantly higher in one of the investigated membranes; this was also corroborated in our evaluation as, after a degradation time of 1.5 h, one group (NCL-E) presented a three times higher collagen degradation than another (NCL-P). In the present study, as well as in the previously mentioned study, bacterial collagenases were used. However, this harsh model might rather simulate a clinical worst-case scenario, as is the case when wound dehiscence occurs rather than uneventful healing. The exposure of collagen membranes to the oral environment during the early healing phase leads to rapid bacterial colonization of the membranes. It has been shown that some bacteria (e.g., *P. gingivalis* or *T. denticola*) are able to resorb collagen membranes, and that bacterial enzymes have the potential to accelerate the degradation of resorbable membranes [27]. Therefore, in the case of wound dehiscence and/or early exposure of membranes, both cross-linked and native collagen membranes may clinically present premature degradation [28,29]. Notably, it should also be mentioned that the used collagenase in this investigation was probably much more potent, in terms of degradation pattern, than the enzymes produced by *A. actinomycetemcomitans*, *T. denticola*, and *P. gingivalis*, as shown by Sela et al. [27], and may therefore reflect a worst-case scenario. The quite early degradation and leakage observed should, therefore, be critically considered and not over-estimated. However, the model was able to test and compare such differences quite impressively, which again does not imply that a seemingly premature loss of occlusivity also means a clinical disadvantage, given the conditions of uneventful healing. However, based on our results, it can be stated that in the case of membrane exposure, a cross-linked collagen membrane may withstand degradation longer than native collagen membranes. 

Previous studies have shown that the cell occlusivity plays a major role in the extent of regeneration. In an animal study in dogs, Polimeni et al. [30] compared cell occlusive ePTFE membranes (pores of 15–25 μm) with macro-porous ePTFE membranes (pores of 300 μm). After a healing time of 8 weeks, it was seen that bone regeneration was significantly higher when membranes with small pores were used. This observation has also been made in several clinical studies. Friedmann et al. have compared the volume preservation of augmented mineralized tissue around dental implants between a native collagen membrane (NCL-P, BioGide) and an enzymatic cross-linked membrane (ECL-B, OSSIX). After 6 months, the amount of mineralized tissue around the implant was significantly higher in the cross-linked membrane group, compared to the native collagen membrane group, both in the vertical and horizontal dimensions [31]. In this context, it should be mentioned that another study using the same set-up observed 50% exposure of the cell occlusive membrane, compared to no exposure of the macro-porous membrane [32]. This might lead to the awareness that porous membranes better support wound healing, as they simplify blood flow and tissue nutrition. Nevertheless, the clinical impact of this finding is questionable. Thus, in a randomized clinical study comparing the performance and adverse events with a native collagen membrane (BioGide) against an enzymatically cross-linked membrane (Ossix Plus), the occurrence of post-op dehiscence was comparable. The dehiscence rate in the cross-linked membrane group was even slightly lower than in the native membrane group [33]. The angiogenesis with different collagen membranes has been investigated by Schwarz et al. [34] in rats. The native collagen membrane (Bio-Gide) showed, after only two weeks, an almost complete and homogenous vascularization. In contrast, the cross-linked collagen membrane (Ossix Plus) showed no signs of angiogenesis after 24 weeks. On the other hand, it is well known that due to their porosity, nutrients can permeate cross-linked collagen membranes in a comparable manner to native collagen membranes, despite their higher cellular barrier function [35]. ECL-B was the only membrane in our study which, even after 12 h of in vitro degradation, was not permeable for the boar spermatozoa. Again, it must be critically mentioned that the amount of degradation does not necessarily go hand in hand with the permeability of cells, as our results indicated. 

## 5. Conclusions

In summary, the considered topic is highly complex, and it remains difficult to draw definitive conclusions, with respect to clinical outcomes, especially when considering results derived from an in vitro study. The clinical behavior of various collagen membranes may differ, as many cellular and non-cellular factors play potential roles in the degradation process. However, as the findings of our study were well in accordance with results of several previous animal studies, it could be concluded that the surrogate model was able to facilitate the rapid and cheap screening of cell occlusivity for different collagen membranes in a very standardized manner. In particular, claims of long degradation resistance can be easily tested and compared. As the boar spermatozoa used in the present report had a size of 9 × 5 μm, smaller bacteria are also likely able to penetrate the leaking membranes; in this regard, our proposed study set-up may provide valuable information, although it must be acknowledged that sperm cells show active mobility and do not only translocate by growth.

## Data Availability

The data presented in this study are available upon request from the corresponding author.
